# Effect of adjuvant radiotherapy on the local recurrence of oral squamous cell carcinoma with perineural invasion: A systematic review

**DOI:** 10.1111/coa.13239

**Published:** 2018-10-30

**Authors:** Jasper Vonk, Kim A. Smit, Jan L. N. Roodenburg, Bert van der Vegt, Gyorgy B. Halmos, Johanna G. M. Vemer‐van den Hoek, Pieter U. Dijkstra, Max J. H. Witjes

**Affiliations:** ^1^ Department of Oral and Maxillofacial Surgery University of Groningen University Medical Center Groningen Groningen The Netherlands; ^2^ Department of Pathology University of Groningen University Medical Center Groningen Groningen The Netherlands; ^3^ Department of Otorhinolaryngology/Head and Neck Surgery University of Groningen University Medical Center Groningen Groningen The Netherlands; ^4^ Department of Radiotherapy University of Groningen University Medical Center Groningen Groningen The Netherlands; ^5^ Department of Rehabilitation University of Groningen University Medical Center Groningen Groningen The Netherlands

**Keywords:** oral cancer, perineural invasion, postoperative radiotherapy, squamous cell carcinoma

## Abstract

**Objectives of the Review:**

The decision whether to include postoperative radiotherapy on patients with oral squamous cell carcinoma depends on the risk of local recurrence. The objectives of this study were to systematically review literature on whether perineural invasion in oral squamous cell carcinoma patients is associated with higher local recurrence rates and whether local recurrence is influenced by the administration of postoperative radiotherapy in patients presenting with perineural invasion.

**Type of Review:**

Systematic review.

**Search Strategy:**

Embase, PubMed, Web Of Science.

**Evaluation Method:**

The databases above were searched for studies that analysed: the treatment of oral squamous cell carcinoma patients with perineural invasion, local recurrence and postoperative radiotherapy. The data of seven studies were analysed qualitatively.

**Results:**

The overall quality of the studies was moderate to low. There was no evidence of the effect of postoperative radiotherapy on local recurrence rates in patients presenting with perineural invasion. Some evidence suggests that local recurrence rates may increase in cases of multifocal perineural invasion, especially if nerves >1 mm are involved but these data should be interpreted with caution due to the low‐quality evidence.

**Conclusions:**

High‐quality evidence regarding the prognostic value of perineural invasion and the impact of postoperative radiotherapy in patients presenting with perineural invasion is lacking in the literature, making it difficult to select a postoperative strategy for early‐stage tumours.

**Keypoints**

- oral cancer
- perineural invasion
- postoperative radiotherapy

## 1 INTRODUCTION

Surgical resection is the primary treatment modality of oral squamous cell carcinoma (OSCC). The decision whether to apply postoperative radiotherapy (PORT) depends on the risk of local or locoregional recurrence (LR).^1^, ^2^, ^3^, ^4^ To improve local control in advanced disease (stage III‐IV), surgical resection is followed by PORT. In early‐stage tumours (I‐II), there is often no indication for radiotherapy. However, PORT should be given to cases with high risk of recurrence such as positive surgical margins (<1 mm), multiple affected lymph nodes (N ≥ 2b) and extracapsular extension, in order to improve locoregional control.^5^ It is not entirely clear in intermediate risk cases of early‐stage OSCC with close margins (1‐5 mm), poor differentiation, pT3‐4, lymphangio invasion and perineural invasion (PNI), as to when PORT should be applied.^5^ There is a need for research regarding adjuvant radiotherapy in intermediate risk cases, which was also pointed out by Blackburn et al.^6^

The risk of LR in the presence of PNI is unclear.^2^, ^7^, ^8^, ^9^, ^10^ Some studies found a significant increase in LR rates in PNI cases,^10^, ^11^, ^12^ while other studies did not.^13^, ^14^ Moreover, it is not clear whether PORT has an additional, positive effect in reducing the local recurrence rate in early OSCC with PNI. Evidence regarding prognosis is needed to justify the role of PORT, because of its side effects such as xerostomia, dysphagia, loss of taste, trismus and osteoradionecrosis.^15^, ^16^, ^17^

The objectives of this study were to systematically review the literature to find whether PNI in OSCC patients is associated with a higher LR rate and whether LR rate is influenced by administration of PORT in OSCC patients presenting with PNI.

## 2 METHODS

### 2.1 Study identification and selection

The study protocol was designed using the PRISMA statement for reporting systematic reviews and meta‐analyses.^18^ A search protocol was developed prior to the study. Studies were sought in electronic databases namely, PubMed, Embase and Web of Science. The last search date was 18 September 2017. No limitations were applied regarding time of study or study design. Only studies written in English were included. A general search strategy was developed together with an information specialist and adapted appropriately to each database (Appendix 1). Publications were included if they described: the treatment of patients with squamous cell carcinoma of the oral cavity; PNI; local recurrence; whether PORT was given or not; and a sample size of ≥10. Local recurrence was defined as histopathologically proven tumour arising within 10 mm from the primary tumour. Publications were excluded if brachytherapy was given, if preoperative radiotherapy or other earlier treatments were provided, if recurrent or secondary tumours were included, if pathological T‐stage was not reported or if the intent of treatment was not curative.

First, titles were assessed for selection by two observers independently (JV and KS). Abstracts from the included titles were then assessed by the same observers. Titles with insufficient information or causing disagreement between the observers were also included for abstract assessment. If an abstract provided insufficient information or disagreement existed between observers, the text was checked. Thereafter, full‐text papers were assessed in a similar way. Finally, the references of the included studies were also perused for inclusion and if any were selected, the same procedure was followed as described above. Studies in which only a part of the study group met the inclusion criteria were included for further analysis of the relevant group. Interobserver agreement was expressed as Cohen's κ and as a percentage of agreement.

In cases of disagreement about inclusion or exclusion, a decision was made by consensus. A third reviewer (MJHW) was consulted to resolve remaining disagreements.

### 2.2 Data extraction

The relevant data of the included papers were extracted onto a standardised form by the first author (JV) according to the following categories: dates over which the study was conducted; patient characteristics; tumour characteristics (including location in the oral cavity, T‐stage and histopathologic factors); adjuvant (chemo)radiotherapy (including chemotherapy agent and dosing, technique of radiotherapy and dose and fractionation of the radiation); and local recurrence (in relation to PNI and adjuvant radiotherapy). All data extraction was verified by another reviewer (PUD).

### 2.3 Study quality assessment

The quality of the included studies was assessed based on the “Newcastle‐Ottawa Scale (NOS) for assessing the Quality of Nonrandomized Studies”^19^ by the first author. These included the study groups, comparability of the groups and ascertainment of outcome of interest. The NOS can be found in Appendix 2.

## 3 RESULTS

### 3.1 Study selection

**Figure 1:**
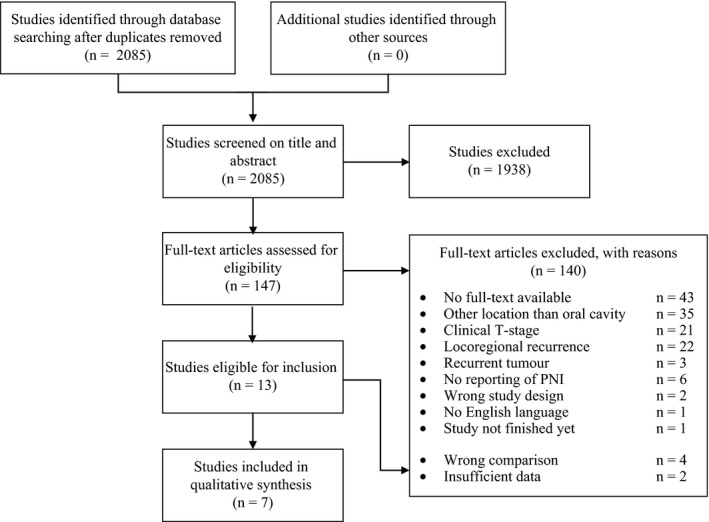
Flow chart of the selection process

The searches in PubMed, Embase and Web of Science yielded 976, 1755 and 63 hits, respectively (Figure 1). After deduplication, 2085 papers remained. The interobserver agreement for title and abstract selection was 0.73 (SE = 0.029), and absolute agreement was 95.7%. Following title and abstract selection, 119 papers remained for full‐text assessment (interobserver agreement 0.61 (SE = 0.089), absolute agreement 97.5%) after which a total of 13 studies were potentially eligible for inclusion in this systematic review. A reference check did not result in additional relevant studies. Six of the 13 studies were excluded because of insufficient data or irrelevant comparisons. Qualitative data analysis was performed on the remaining seven studies. Data synthesis was not performed because of the heterogeneity of the study designs and populations.

### 3.2 Characteristics of included studies

All studies were retrospective cohorts published between 2011 and 2016. Sample size ranged from 78 to 442 patients (Table 1). All the studies included primary tumours of the oral cavity with surgery as a primary treatment. The definition of PNI differed between the studies. The most used definition of PNI was the presence of tumour cells in any of the three layers of the nerve sheath and/or tumour cells in close proximity to the nerve involving more than one‐third of its circumference.^1^, ^8^, ^20^ In another study, the definition of PNI was limited to the presence of tumour cells within any of the three layers of the nerve sheath^21^ and another three studies did not define PNI at all.^22^, ^23^ Three studies included patients with all pathological T‐stages,^1^, ^21^, ^24^ whereas four studies included patients solely with pT1‐2 tumors.^22^, ^23^, ^25^ Two studies excluded patients with positive or close resection margins.^1^, ^23^ All the studies reported the resection margins, but only five studies defined them.^1^, ^20^, ^23^, ^24^, ^25^ Other histopathologic features were described adequately in five studies.^8^, ^20^, ^21^, ^22^, ^25^ One study excluded patients with adverse histopathologic factors such as extracapsular spread and lymphangio invasion.^1^ In five studies, patients were treated with PORT, but no specific indication regarding PNI was reported.^1^, ^8^, ^20^, ^21^, ^23^ Of those studies, four reported that PORT^1^, ^8^, ^20^, ^23^ was applied to 3.6%‐43.4% of the patients. One study did not report which patients were treated with PORT.^21^ Two other studies excluded the patients treated with PORT.^22^, ^25^ All the studies analysed different variables, but all had local recurrence as either a primary or secondary outcome.

**Table 1:** Study characteristics

Author, publication year	Tai, 2011^24^	Ganly, 2012^19^	Chen, 2013^20^	Chatzistefanou, 2014^1^	Aivazian, 2015^7^	Matsushita, 2015^18^	Low, 2016^22^
Country	Taiwan	USA	Taiwan	USA	Australia	Japan	Australia
Center	Taipei Veterans General Hospital	Multicenter^c^	National Taiwan University Hospital	University of Maryland	Royal Prince Alfred Hospital	Nagasaki University	Sydney Head and Neck Cancer Institute
Design	RC	RC	RC	RC	RC	RC	RC
Study period	2001‐2009	1985‐2005	2004‐2009	2005‐2011	1995‐2010	2001‐2011	1988‐2013
Data source	Medical records	NR	Medical records &Pathologic reports	NR	Medical records	Medical records	NR
Total sample (n)	307	164	442	78	318	89	121
Male (n)	267	90	374	55	194	50	75
Age (range)	≤54, n = 172	<60, n = 98	≤50, n = 207	<60, n = 39	64^a^ (30‐92)	>63, n = 48	61^a^ (47‐74)
	>54, n = 135	≥60, n = 66	>50, n = 235	≥60, n = 39		≤63, n = 41	
Follow‐up (mo)	49.1^a^	66^a^	46^b^	42.7^b^	32.4^a^	49.4^b^	38^a^
Treatment							
Surgery	245	164	426	48	180	NR	121
Surgery + RT	22	0	16	30	124	NR	0
Surgery + CRT	40	0	0	0	14	NR	0
Type of RT (dose)	NR (62.8 Gy PNI + 60 Gy PNI−)	—	NR	NR (50‐70 Gy)	NR	NR	—
Type of chemotherapy	Cisplatin	—	—	—	NR	—	—
NOS rating	5	5	5	5	8	6	6

CRT, chemoradiotherapy; n, number; NOS, Newcastle‐Ottawa Scale; NR, not reported; RC, retrospective cohort; RT, radiotherapy.

a Median.

b Mean.

c Memorial Sloan‐Kettering Cancer Center (New York, NY) and Princess Margaret Cancer Center (Toronto, Ontario, Canada).

### 3.3 Local recurrence

None of the seven studies reported PNI as a significant prognostic factor for LR; however, one study reported PNI as a significant prognostic factor if it was presenting multifocally, especially if nerves >1 mm were involved (*P* = 0.049).^8^ None of the seven included studies evaluated the impact of PORT in patients presenting with PNI. A summary of study findings can be found in Table 2.

**Table 2:** Summary of study findings

Author, year	Total, n	pT1	pT2	pT3	pT4	Local recurrence in PNI+ patients			Local recurrence in PNI− patients			HR	*P*‐value
						PNI+ (n)	LR (n)	LR (%)	PNI− (n)	LR (n)	LR (%)		
Tai, 2011	307	146	161	—	—	84	16	19.0%	223	30	13.5%	—	0.221
Ganly, 2012	164	76	88	—	—	22^b^	1	4.5%	124^b^	13	10.5%	—	0.56
Chen, 2013	442	272	170	—	—	65	12	18.5%^c^	377	55	14.6%	—	NS
Chatzistefanou, 2014	78	50^a^		28^a^		—	—	26.3%^d^	—	—	27.5%^d^	—	0.332
Aivazian, 2015	318	108	106	26	76	—	—	—	—	—	—	1.26^e^	1.00
Matsushita, 2015	89	82^a^		7^a^		—	—	23%	—	—	26%	—	NS
Low, 2016	121	121	—	—	—	24	5	20.8%	65	6	9.2%	—	0.33

HR, hazard ratio; LR, local recurrence; n, number; NS, not significant; PNI−, perineural invasion‐negative; PNI+, perineural invasion‐positive.

a These numbers represent T1 and T2 or T3 and T4 cases.

b There were also a number of patients in which PNI was not reported.

c PNI and lymphovascular invasion were combined as a high risk group in the calculations of local recurrence.

d Local recurrence rates were calculated in patients who did not receive postoperative radiotherapy.

e Hazard ratios were 0.42 (*P* = 0.247) and 2.24 (*P* = 0.049) for unifocal and multifocal, respectively.

## 4 DISCUSSION

### 4.1 Summary of main results

We used a systematic review to investigate the impact of PNI on LR in OSCC patients and whether LR is influenced by the administration of PORT. Only seven studies could be included, and these papers only partly answered the research questions; therefore, high‐quality evidence regarding the impact of PNI on LR rates in patients with OSCC is lacking. All studies reported that there was no significant difference in LR between patients presenting with and without PNI. However, one study reported that PNI was a significant prognostic factor when it is multifocal, especially if nerves >1 mm are involved. None of the included studies reported the impact of PORT on LR in patients presenting with PNI.

### 4.2 Comparison with other reviews

An earlier systematic review reported that PNI is not a significant prognostic factor for locoregional recurrence.^26^ That systematic review included studies describing squamous cell carcinoma in the complete head and neck area and studies only reporting clinical T‐stage were not excluded. Also, local and regional recurrences were listed as locoregional recurrence irrespective of whether these types of recurrences had other aetiologies. Our aim was to evaluate the impact of PNI on LR more precisely by excluding studies only reporting clinical T‐stage and those only including OSCC. A partially retrospective and partially prospective study reported PNI as an independent predictor of LR if nerves >1 mm were involved^10^; however, this study was excluded in our study because pathological T‐stage was not described and squamous cell carcinoma of the pharynx was also included. Another study did not find any association between nerve size and LR; however, this might be explained by the fact that only nerves smaller than 1 mm were encountered in that study.^9^ The extent of PNI was evaluated by Chinn et al. on attempting to demonstrate an association between the extent of PNI and the size of the nerves involved, but they failed, probably due to the lack of an adequate sample size (n = 20).^7^

### 4.3 Overall completeness and applicability of evidence

The major limitation of the current literature is the lack of a standardised definition of PNI. Based on the results of the present systematic review, there is a need for a standardised definition of PNI in order to obtain exact numbers of its incidence and to evaluate the association between PNI and prognosis.

Furthermore, the majority of the included studies did not describe the location, size of the involved nerves and the extent of PNI. Aivazian et al^8^ reported a clinically significant difference between the prognostic value of unifocal and multifocal PNI, especially combined with invasion of nerves >1 mm; therefore, it seems it is not enough to only report PNI as absent or present.

Finally, there were no data available on the selection criteria of patients receiving adjuvant treatment because of PNI. Therefore, it remains unclear at which point PORT was administered to the PNI‐positive and the PNI‐negative group and no conclusions can be drawn about the additional effect of PORT on LR.

### 4.4 Quality of evidence

Although over 2000 papers were identified in the database search, only seven papers could be included in this systematic review. A very common reason for exclusion was the non‐reporting of pathological T‐stage; only clinical T‐stage was reported. Pathological T‐stage is essential to evaluate prognosis. Furthermore, pT1‐2 tumours are clinically most important because an indication for PORT is based on secondary histopathological factors in these early‐stage tumours, whereas most institutes use pT3‐4 tumours as an indication for PORT.^5^ Also a considerable number of studies were excluded because locoregional recurrence was reported instead of separate local and regional recurrence. The level of evidence of the included studies was limited because of their retrospective design.

### 4.5 Implications for research

In order to determine the indication for PORT, prospective studies need to be performed to investigate the effect of PORT in patients presenting with PNI on LR. It would be important to introduce a standardised definition of PNI to obtain exact incidence rates. Also, the extent of PNI should be described more specifically by means of the location and the size of the involved nerves. Finally, accurate data registration including precise description of the cohort, pathological T‐ and N‐stages, resection margins, local recurrence and other pathological tumour characteristics (such as depth of invasion, pattern of growth and lymphovascular invasion) would be essential in order to evaluate prognosis.

## 5 CONCLUSION

Based on the available evidence, it is not clear whether there is an indication for PORT in unifocal PNI; moreover, high‐quality evidence is lacking on the impact of PORT in OSCC patients presenting with PNI.

## CONFLICT OF INTEREST

There are no conflicts of interest to declare.

## APPENDIX 1 Search strategy

### PubMed

(“Mouth”[Mesh] OR “Mouth Neoplasms”[Mesh] OR tongue[tiab] OR oral[tiab] OR mouth[tiab] OR gingiva*[tiab])

AND (“Mouth Neoplasms”[Mesh] OR OSCC[tiab] OR SCC[tiab] OR squamous cell[tiab] OR cancer[tiab] OR carcinoma*[tiab] OR tumour*[tiab] OR tumor*[tiab])

AND (“Radiotherapy, Adjuvant”[Mesh] OR “Chemoradiotherapy”[Mesh] OR “radiotherapy” [Subheading] OR chemoradiother*[tiab] OR chemo radio therap*[tiab] OR radio chemo therap*[tiab] OR radiochemother*[tiab] OR (chemoradio*[tiab] AND therap*[tiab]) OR (radiochemo*[tiab] AND therap*[tiab]) OR radiother*[tiab] OR (radiation[tiab] AND (postoperative[tiab] OR post‐operative[tiab] OR adjuvant[tiab])))

AND (“Neoplasm Recurrence, Local”[Mesh] OR “Mortality”[Mesh] OR “Survival”[Mesh] OR recurren*[tiab] OR surviv*[tiab] OR mortality[tiab] OR death*[tiab])

AND (“Neoplasm Invasiveness”[Mesh] OR Perineural[tiab] OR invasi*[tiab] OR characteristic*[tiab] OR agressi*[tiab])

### Embase

(‘Mouth’/exp OR ‘mouth cancer’/exp OR ‘tongue tumor’/exp OR tongue:ab,ti OR oral:ab,ti OR mouth:ab,ti OR gingiva*:ab,ti)

AND

(‘mouth cancer’/exp OR ‘tongue tumor’/exp OR oscc:ab,ti OR scc:ab,ti OR ‘squamous cell’:ab,ti OR cancer:ab,ti OR carcinoma*:ab,ti OR tumour*:ab,ti OR tumor*:ab,ti)

AND

(‘cancer adjuvant therapy’/exp OR ‘cancer radiotherapy’/exp OR chemoradiother*:ab,ti OR ((chemoradio* OR radiochemo*) NEXT/1 therap*):ab,ti OR radiochemother*:ab,ti OR radiother*:ab,ti OR (chemo* NEAR/3 radio* NEAR/3 therap*):ab,ti OR (radiation AND (postoperative OR ‘post operative’ OR adjuvant)):ab,ti)

AND

(‘cancer mortality’/exp OR ‘cancer recurrence’/exp OR ‘cancer survival’/exp OR recurren*:ab,ti OR surviv*:ab,ti OR mortality:ab,ti OR death*:ab,ti)

AND

(‘tumor invasion’/exp OR perineural:ab,ti OR invasi*:ab,ti OR characteristic*:ab,ti OR agressi*:ab,ti)

### Web of Science

TS=(“mouth” OR “tongue” OR gingiva* OR “oral”)

AND

TS=(cancer* OR tumour* OR tumor* OR carcinoma* OR neoplasm* OR “scc” OR “oscc” OR “squamous cell”)

AND

(TS=(chemo* NEAR/3 radio* NEAR/3 therap*) OR TS=(chemoradio* OR radiochemo* OR radiotherap*) OR TS=(“radiation” AND (“postoperative” OR “post operative” OR “adjuvant”)))

AND

TS=(“mortality” OR surviv* OR death* OR recurr*)

AND

TS=(invas* OR characteristic* OR agressi* OR “perineural”)

## APPENDIX 2

### Newcastle—Ottawa quality assessment scale case control studies

Note: A study can be awarded a maximum of one star for each numbered item within the Selection and Exposure categories. A maximum of two stars can be given for Comparability.

#### Selection

1 Is the case definition adequate?
  - yes, with independent validation *
  - yes, eg record linkage or based on self reports
  - no description
2 Representativeness of the cases
  - consecutive or obviously representative series of cases *
  - potential for selection biases or not stated
3 Selection of Controls
  - community controls *
  - hospital controls
  - no description
4 Definition of Controls
  - no history of disease (endpoint) *
  - no description of source

#### Comparability

1. Comparability of cases and controls on the basis of the design or analysis
  - study controls for______________ (Select the most important factor.) *
  - study controls for any additional factor * (This criteria could be modified to indicate specific control for a second important factor.)

#### Exposure

1 Ascertainment of exposure
  - secure record (eg surgical records) *
  - structured interview where blind to case/control status *
  - interview not blinded to case/control status
  - written self report or medical record only
  - no description
2 Same method of ascertainment for cases and controls
  - yes *
  - no
3 Non‐Response rate
  - same rate for both groups *
  - non respondents described
  - rate different and no designation

### Newcastle—Ottawa quality assessment scale cohort studies

Note: A study can be awarded a maximum of one star for each numbered item within the Selection and Outcome categories. A maximum of two stars can be given for Comparability

#### Selection

1 Representativeness of the exposed cohort
  - truly representative of the average _______________(describe) in the community *
  - somewhat representative of the average ______________in the community *
  - selected group of users eg nurses, volunteers
  - no description of the derivation of the cohort
2 Selection of the non exposed cohort
  - drawn from the same community as the exposed cohort *
  - drawn from a different source
  - no description of the derivation of the non exposed cohort
3 Ascertainment of exposure
  - secure record (eg surgical records) *
  - structured interview *
  - written self report
  - no description
4 Demonstration that outcome of interest was not present at start of study
  - yes *
  - no

#### Comparability

1. Comparability of cohorts on the basis of the design or analysis
  - study controls for _____________(select the most important factor) *
  - study controls for any additional factor * (This criteria could be modified to indicate specific control for a second important factor.)

#### Outcome

1 Assessment of outcome
  - independent blind assessment *
  - record linkage *
  - self report
  - no description
2 Was follow‐up long enough for outcomes to occur
  - yes (select an adequate follow up period for outcome of interest) *
  - no
3 Adequacy of follow up of cohorts
  - complete follow up—all subjects accounted for *
  - subjects lost to follow up unlikely to introduce bias—small number lost ‐ > _____________ % (select an adequate %) follow up, or description provided of those lost) *
  - follow up rate <_____________% (select an adequate %) and no description of those lost
  - no statement
